# Supplementary material for: Standard Visual and Ordinal Coronary Calcium Scoring on PET/CT: Agreement with Agatston Scoring and Prognostic Implications
Source: Diagnostics (Basel). 2025 Nov 22;15(23):2969. doi: 10.3390/diagnostics15232969 (PMC12691459; doi:10.3390/diagnostics15232969)
Supplement: Supplementary file 1 [file diagnostics-15-02969-s001.zip › Supplementary Mateterial S1. Visual Scale Instruction.pdf]

# Instruction for Visual Scale

**Study Aim**

This study evaluates the concordance between visual scoring of coronary artery calcium (CAC) on PET/CT and the gold-standard Agatston score categories (0, 1–99, 100–399, ≥ 400) obtained from EKG-gated chest CT performed within three months.

---

**What to Do**

1. **Open the "visual" sheet** in the attached Excel file.
2. For each case, assign a severity score to the CAC seen on:
   - the low-dose CT component of the PET/CT, and
   - the corresponding CCTA.
3. Use the following 4-point scale:
   - **0** = None
   - **1** = Mild
   - **2** = Moderate
   - **3** = Severe

**Special Cases**

- If the patient has undergone **PCI** (percutaneous coronary intervention) or **CABG** (coronary artery bypass grafting), confirm this in the EMR.
  - Do **not** score CAC for that vessel.
  - Instead, enter **"PCI"** or **"CABG"** in the cell.

---

**Reference Example**

See Figure 2 in the attached paper (**Fresno et al. *AJR Am J Roentgenol.* 2022 Oct;219:569-578**) for an example of visual scoring.

---

**Practical Tips**

- **Right coronary artery (RCA):** Take care not to mistake valvular calcification for coronary artery calcification.
- If you are unsure of the coronary anatomy, review available coronary CT angiography in PACS before scoring.

---
