# Supplementary material for: Standard Visual and Ordinal Coronary Calcium Scoring on PET/CT: Agreement with Agatston Scoring and Prognostic Implications
Source: Diagnostics (Basel). 2025 Nov 22;15(23):2969. doi: 10.3390/diagnostics15232969 (PMC12691459; doi:10.3390/diagnostics15232969)
Supplement: Supplementary file 1 [file diagnostics-15-02969-s001.zip › Supplementary Mateterial S2. Ordinal Scale Instruction.pdf]

Instruction for Ordinal Scale

Study Aim

This study evaluates the concordance between visual scoring of coronary artery calcium (CAC) on PET/CT and the gold-standard Agatston score categories (0, 1–99, 100–399, ≥ 400) obtained from EKG-gated chest CT performed within three months.

Where to Record

Enter the ordinal scores in the **“ordinal” sheet** in the attached Excel file.

Target Vessels

- LM (left main)
- LAD (left anterior descending)
- LCX (left circumflex)
- RCA (right coronary artery)

Scoring Rules

Grade each vessel according to the percentage of its length occupied by calcified plaque:

Vessel involvement	Category	Score
0 %	Absent	**0**
1–24 %	Mild	**1**
25–49 %	Moderate	**2**
≥ 50 %	Severe	**3**

Excel tip: When you enter a score (0–3) for each vessel, the sheet automatically sums the total (0–12).

Special Cases

- If the patient has undergone **PCI** (percutaneous coronary intervention) or **CABG** (coronary artery bypass grafting), confirm this in the EMR.
    - Do **not** score CAC for that vessel.
    - Instead, enter **“PCI”** or **“CABG”** in the cell.

Defining Vessel Length

If the vessel boundaries are unclear, use these landmarks:

**Vessel**	**From**	**To**
**LM**	Aortic origin	Bifurcation into LAD and LCX
**LAD**	Bifurcation	Approximately cardiac apex
**LCX**	Bifurcation	Origin of the PDA (before it meets the RCA)
**RCA**	Aortic origin	Origin of the PDA

*The posterior descending artery (PDA) itself is **not** scored.*

Refer to the attached schematic of the LAD, LCX, and RCA if needed (Kubicka et al. *Radiographics.* 1986;6:661-701. Figure 1–5).

Total Score and Global Category

With four vessels, the total score ranges from **0 to 12**:

**Total score**	**Global category**
0	Absent
1–3	Mild
4–6	Moderate
≥ 7	Severe

Methodological Basis

This protocol merges two published approaches:

**Reference**	**Vessels included**	**Length thresholds**
**Shemesh et al.**	LM, LAD, LCX, RCA	< 1/3, 1/3–2/3, ≥ 2/3
**Choi et al.**	LAD, LCX, RCA	1–24 %, 25–49 %, ≥ 50 %

We retained the four-vessel scheme from **Shemesh et al. (*Radiology.* 2010;257:541-8)** and the percentage cut-offs from **Choi et al. (*Circulation.* 2023;148:1154-1164)** to apply a more sensitive grading system; visual or ordinal scales often underrate severity compared with Agatston scoring. For visual examples of mild, moderate, and severe involvement using the Shemesh method, see Figure 2 in the attached paper (**Chiles et al. *Radiology.* 2015;276:82-90).**
